# Supplementary material for: Clinical performance of different SARS‐CoV‐2 IgG antibody tests
Source: J Med Virol. 2020 Jun 19;92(10):2243–7. doi: 10.1002/jmv.26145 (PMC7300776; doi:10.1002/jmv.26145)
Supplement: Supplementary file 1 — Supporting information [file JMV-92-2243-s002.doc]

TABLE S1 – For sensitivity tested individual follow-up samples of SARS-CoV-2 PCR-confirmed individuals at different time points and generated results.

| **Sample Nr.** | **Day after confirmed SARS-CoV-2 PCR** | **Euroimmun (ELISA) Ratio** | **Vircell (ELISA)**  **Index** | **IFA (in-house) qual.** | **Assure Tech (Rapid Test) qual.** | **PRNT Titer** |
| --- | --- | --- | --- | --- | --- | --- |
| 1 | 5 | neg. | 1.0 | neg. | neg. | neg. |
| 2 | 6 | 6,2 | 3.6 | pos. | pos. | 1:160 |
| 3 | 6 | neg. | neg. | neg. | neg. | neg. |
| 4 | 6 | neg. | 1.1 | pos. | pos. | 1:40 |
| 5 | 6 | 11 | 3.3 | pos. | pos. | 1:1280 |
| 6 | 6 | 10.1 | 4.1 | pos. | pos. | 1:640 |
| 7 | 7 | 15.8 | 4.2 | pos. | pos. | 1:640 |
| 8 | 7 | 12.3 | 5.1 | pos. | pos. | 1:1280 |
| 9 | 7 | 14.1 | 5.1 | pos. | pos. | 1:1280 |
| 10 | 8 | neg. | neg. | neg. | neg. | neg. |
| 11 | 8 | neg. | neg. | neg. | neg. | neg. |
| 12 | 8 | neg. | neg. | pos. | neg. | 1:40 |
| 13 | 8 | 10 | 5 | pos. | pos. | 1:640 |
| 14 | 8 | 14.5 | 5.1 | pos. | pos. | 1:640 |
| 15 | 8 | 13.8 | 5.1 | pos. | - | 1:320 |
| 16 | 9 | neg. | neg. | pos. | neg. | 1:10 |
| 17 | 9 | 15.7 | 4.9 | pos. | pos. | 1:1280 |
| 18 | 10 | 1.13 | 2.7 | pos. | pos. | 1:80 |
| 19 | 10 | neg | 1.3 | pos. | neg. | 1:80 |
| 20 | 10 | 5.2 | 2.5 | pos. | pos. | 1:320 |
| 21 | 10 | 6.4 | 1.2 | pos. | pos. | 1:640 |
| 22 | 10 | 16.2 | 4.4 | pos. | pos. | 1:1280 |
| 23 | 11 | 17 | 5.1 | pos. | pos. | 1:640 |
| 24 | 13 | 16.6 | 5.1 | pos. | pos. | 1:1280 |
| 25 | 13 | 15.6 | 4.4 | pos. | pos. | 1:160 |
| 26 | 14 | 6.14 | 4.6 | pos. | pos. | 1:320 |
| 27 | 14 | 17 | 5.1 | pos. | pos. | 1:1280 |
| 28 | 16 | 7.2 | 4.8 | pos. | pos. | 1:640 |
| 29 | 16 | 16.2 | 5.1 | pos. | pos. | 1:640 |
| 30 | 16 | 14.8 | 5.1 | pos. | pos. | 1:640 |
| 31 | 17 | 14.8 | 3.7 | pos. | pos. | 1:640 |
| 32 | 17 | 13.4 | 3.9 | pos. | pos. | 1:1280 |
| 33 | 18 | 13 | 5.1 | pos. | pos. | 1:1280 |

Euroimmun (Ratio <0.8 = negative, 0.8-<1.1 = equivocal, ≥ 1.1 = positive), Vircell (Index <0.4 = neg., 0.4-0.6 = equivocal, >0.6 = pos.); pos., positive; neg., negative; -, not tested.
