# Supplementary material for: Clinical performance of different SARS‐CoV‐2 IgG antibody tests
Source: J Med Virol. 2020 Jun 19;92(10):2243–7. doi: 10.1002/jmv.26145 (PMC7300776; doi:10.1002/jmv.26145)
Supplement: Supplementary file 2 — Supporting information [file JMV-92-2243-s001.doc]

TABLE S2 – For specificity tested follow-up samples of individuals with selected PCR- or serologically-confirmed infections and generated results.

| **Sample Nr.** | **Recently PCR-/serologically-confirmed infected with** | **Euroimmun (ELISA) Ratio** | **Vircell (ELISA) Index** | **IFA (in-house)**  **qual.** | **Assure Tech (Rapid Test)**  **qual.** |
| --- | --- | --- | --- | --- | --- |
| 1 | HCOV-OC43 | neg. | neg. | neg. | neg. |
| 2 | HCOV-OC43 | 0.9 | neg. | neg. | neg. |
| 3 | HCoV-OC43 | neg. | neg. | neg. | neg. |
| 4 | HCoV-OC43 | neg. | neg. | neg. | neg. |
| 5 | HKU 1 | neg. | neg. | neg. | neg. |
| 6 | SARS-CoV-1 | neg. | 2.2 | pos. | - |
| 7 | SARS-CoV-1 | neg. | 3.8 | pos. | - |
| 8 | SARS-CoV-1 | neg. | 3.9 | pos. | - |
| 9 | SARS-CoV-2 neg. | neg. | neg. | neg. | - |
| 10 | SARS-CoV-2 neg. | neg. | neg. | neg. | - |
| 11 | SARS-CoV-2 (neg.) | neg. | neg. | - | - |
| 12 | SARS-CoV-2 + Multiplex* neg. | neg. | neg. | neg. | - |
| 13 | HCoV-229E | neg. | neg. | neg. | - |
| 14 | HCoV-229E | neg. | 1.5 | neg. | - |
| 15 | HCoV 229E + Parainfluenza Virus Type 3 | neg. | neg. | neg. | neg. |
| 16 | HCoV-229E | neg. | neg. | neg. | neg. |
| 17 | HCoV-229E | neg. | neg. | neg. | neg. |
| 18 | HCoV-NL63 + Entero-/Rhinovirus | neg. | neg. | neg. | neg. |
| 19 | HCoV-NL63 | neg. | neg. | - | - |
| 20 | CMV (+ IgM antibody pos.) | neg. | neg. | neg. | neg. |
| 21 | CMV (+IgM antibody pos.) | neg. | neg. | neg. | neg. |
| 22 | CMV (+ IgM antibody pos.) | neg. | neg. | - | - |
| 23 | EBV-VCA-IgM pos. | neg. | neg. | neg. | neg. |
| 24 | EBV (+ -VCA-IgM antibody pos.) | neg. | neg. | neg. | neg. |
| 25 | EBV-VCA-IgM antibody pos. | neg. | - | neg. | - |
| 26 | EBV-VCA-IgM antibody pos. | neg. | - | unsp. | - |

Euroimmun (Ratio <0.8 = negative, 0.8-<1.1 = equivocal, ≥ 1.1 = positive); Vircell (Index <0.4 = neg., 0.4-0.6 = equivocal, >0.6 = pos.); pos., positive; neg., negative; unsp., unspecific; *Biofire® Filmarray® 20 Target Respiratory Panel (bioMérieux, Nürtingen, Baden-Württemberg, Germany); -, not tested.
